# The association of long-term glycaemic variability versus sustained chronic hyperglycaemia with heart rate-corrected QT interval in patients with type 2 diabetes

**DOI:** 10.1371/journal.pone.0183055

**Published:** 2017-08-28

**Authors:** Jian-bin Su, Xiao-hua Yang, Xiu-lin Zhang, Hong-li Cai, Hai-yan Huang, Li-hua Zhao, Feng Xu, Tong Chen, Xing-bo Cheng, Xue-qin Wang, Yan Lu

**Affiliations:** 1 Department of Endocrinology, The First Affiliated Hospital of Soochow University, Suzhou, China; 2 Department of Endocrinology, The Second Affiliated Hospital of Nantong University, Nantong, China; 3 Department of Endocrinology, The Affiliated Haian Hospital of Nantong University, Haian, China; 4 Department of Clinical Laboratory, The Second Affiliated Hospital of Nantong University, Nantong, China; 5 Department of Geriatrics, The Second Affiliated Hospital of Nantong University, Nantong, China; Shanghai Diabetes Institute, CHINA

## Abstract

**Objectives:**

Prolonged heart rate-corrected QT(QTc) interval is related to ventricular arrhythmia and cardiovascular mortality, with considerably high prevalence of type 2 diabetes. Additionally, long-term glycaemic variability could be a significant risk factor for diabetic complications in addition to chronic hyperglycaemia. We compared the associations of long-term glycaemic variability versus sustained chronic hyperglycaemia with the QTc interval among type 2 diabetes patients.

**Methods:**

In this cross-sectional study, 2904 type 2 diabetes patients were recruited who had undergone at least four fasting plasma glucose (FPG) and 2-hour postprandial plasma glucose (PPG) measurements (at least once for every 3 months, respectively) during the preceding year. Long-term glycaemic variabilities of FPG and 2-hour PPG were assessed by their standard deviations (SD-FPG and SD-PPG, respectively), and chronic fasting and postprandial hyperglycaemia were assessed by their means (M-FPG and M-PPG, respectively). HbA1c was also determined upon enrolment to assess current overall glycaemic control. QTc interval was estimated from resting 12-lead electrocardiograms, and more than 440 ms was considered abnormally prolonged.

**Results:**

Patients with prolonged QTc interval (≥440 ms) had greater M-FPG, M-PPG, SD-PPG and HbA1c than those with normal QTc interval but comparable SD-FPG. QTc interval was correlated with M-FPG, M-PPG, SD-PPG and HbA1c (*r* = 0.133, 0.153, 0.245 and 0.207, respectively, *p* = 0.000) but not with SD-FPG (*r* = 0.024, *p* = 0.189). After adjusting for metabolic risk factors via multiple linear regression analysis, SD-PPG, M-PPG and HbA1c (*t* = 12.16, 2.69 and 10.16, respectively, *p* = 0.000) were the major independent contributors to the increased QTc interval. The proportion of prolonged QTc interval increased significantly from 10.9% to 14.2% to 26.6% for the first (T1) to second (T2) to third (T3) tertiles of SD-PPG. After adjusting via multiple logistic regression analysis, the odd ratios of prolonged QTc interval of the T2 and T3 versus the T1 of SD-PPG were 1.15 (95% CI, 0.82–1.60) and 2.62 (1.92–3.57), respectively.

**Conclusions:**

Increased long-term variability of PPG is a strong independent risk factor for prolonged QTc interval in type 2 diabetes patients, in addition to long-term postprandial hyperglycaemia and current HbA1c.

## Introduction

The electrocardiographic QT interval reflects the total time taken for ventricular myocardial depolarization (QRS complex) and repolarization (T wave) [1]. A prolonged heart rate-corrected QT interval (prolonged QTc interval) not only may impart ventricular arrhythmia but is also associated with increased all-cause and cardiovascular mortality in type 2 diabetes [2, 3]. A prolonged QTc interval has been shown to be related to various cardiometabolic risks, such as obesity [4], hypertension [5], lipid disorders [6], hyperglycaemia [7], hyperinsulinaemia [8], and so on. The prevalence of prolonged QTc interval is considerably high among type 2 diabetes [9], a disease that is hallmarked by glycaemic disorders. Therefore, the prolonged QTc interval may be mainly or partly dependent on glycaemic disorders in type 2 diabetes.

Glycaemic disorders in diabetes are not only limited to fasting plasma glucose (FPG) and postprandial plasma glucose (PPG) but can be extended to glycaemic variability [10]. In addition to sustained hyperglycaemia, glycaemic variability could be an significant risk factor for vascular complications in diabetes [11]. The roles of the various glycaemic parameters that participate in the development of diabetic complications may differ [12–14]. Furthermore, diabetic complications depend both on the duration of diabetes and the degree of glycaemic disorders [15]. Therefore, we hypothesized that long-term glycaemic variability may play an essential role in the development of diabetic complications, such as a prolonged QTc interval. Long-term glycaemic variability parameters can be assessed based on the annual variability of FPG and PPG. Annual variation in FPG was reported to be a strong predictor of diabetic nephropathy [16], diabetic retinopathy [17], ischaemic stroke [18], and all-cause and cardivascular mortality in type 2 diabetes patients [19]. However, few studies thus far have evaluated the relationship between the time-dependent variation in PPG and cardiovascular complications among diabetic patients, such as prolonged QTc interval.

The present study is designed to compare the associations of long-term glycaemic variability, especially long-term PPG variation, versus sustained chronic hyperglycaemia with the QTc interval in a large population of type 2 diabetes patients.

## Methods

### Ethics statement

The study was approved by the Medical Ethics Committee of the Second Affiliated Hospital of Nantong University. And informed consent in writing was obtained from each participant.

### Study design and participants

This cross-sectional study was performed in patients with type 2 diabetes who were followed up at the department of Endocrinology of the Second Affiliated Hospital of Nantong University between January 2011 and July 2016. Inclusion criteria were (1) diagnosis of type 2 diabetes according to the criteria of ADA in 2011 [20]; (2) at least four FPG and 2-hour PPG measurements (at least once for every 3 months, respectively) during the 1 year before enrolment; and (3) had received current hypoglycaemic treatments for more than 3 months. In addition, exclusion criteria were (1) type 1 diabetes, or testing positive for the antibody of glutamic acid decarboxylase and insulin; (2) cardiac arrhythmias such as fibrillation and flutter; (3) cardiac conduction block such as atrio-ventricular and bundle-branch blocks; (4) heart valve disease, myocardial infarction, or heart surgery; (5) taking any drugs could affect the QT interval such as tricyclic antidepressant; (6) chronic hepatic disease and kidney disease or previous malignancy; (7) acute complications including infections and diabetic ketoacidosis, etc.; and (8)other endocrine disorders may have effect on glycaemic metabolism such as hypothyroidism and hyperthyroidism. Finally, a total of 2904 patients with a median diabetic duration of 4.0 years were enrolled in the study. S1 STROBE Checklist had provided the STROBE checklist for the cross-sectional study.

### Baseline data collection

Upon enrolment, all participants were interviewed by trained investigators to record their age, sex, medical history, medication use (hypoglycaemic treatments, hypertensive treatment and statins medication) and health behaviours (smoking). The hypoglycaemic treatments included lifestyle intervention alone, insulin, insulin-secretagogues and insulin-sensitizers. Systolic blood pressure (SBP), diastolic blood pressure (DBP) and body mass index (BMI) were examined for further analysis. Those with SBP ≥140 mmHg, with DBP ≥90 mmHg, or receiving antihypertensive agents were considered as hypertensive.

### Estimating of glycaemic parameters

The FPG and 2-hour PPG values of the participants (at least once for every 3 months, respectively) recorded during the 1 year preceding enrolment were available from the HIS. Long-term glycaemic variabilities of FPG and 2-hour PPG were assessed by their standard deviations (SDs), i.e., SD-FPG and SD-PPG, respectively. Long-term average glucose parameters were assessed by their means, i.e., M-FPG and M-PPG, respectively. HbA1c was also determined upon enrolment to assess current overall glycaemic control.

### Laboratory examination

Fasting blood samples were also drawn for the further biochemical tests. Serum insulin level (miu/L) was determined using magnetic beads-based enzymatic spectrofluorometric immunoassay (AIA360, TOSOH). Plasma glucose level was determined using the glucose oxidase method (Model 7600 Series, Hitachi). Index of homeostasis model assessment was used to estimate insulin resistance (HOMA-IR). HbA1c values were determined by the method of high-performance liquid chromatography in the Bio-Rad system. Serum uric acid (UA), total cholesterol (TC), high-density lipoprotein(HDL) cholesterol, low-density lipoprotein(LDL) cholesterol and triglycerides (TG) were measured by an automatic analyser (Model 7600 Series, Hitachi).

### QTc interval from electrocardiogram(ECG)

Standard resting 12-lead ECGs (FX-7402, CardiMax, FuTian Beijing Ltd., China) was performed for all participants. The ECG from each participant was recorded on a standard paper with a waveforms-amplitude of 10 mm/mV and a travelling-rate of 25 mm/s. The QT and RR intervals were calculated based on ten consecutive R waves on the ECG. The QT interval was defined as the duration from the beginning of the QRS complex to the end of the T wave [21]. The beginning of the QT interval was defined as the first negative deflection of the QRS complex, and the end was defined as the point that slope of T wave merged with the baseline [1,21]. The QT interval was corrected with the RR interval using Bazett’s formula, where QTc(ms) = QT/square root of RR(seconds) [21]. A QTc interval more than 440 ms was considered abnormally prolonged [9].

### Statistical analyses

Clinical variables were calculated for the total participants and subgroups (normal QTc interval versus prolonged QTc interval). Normally distributed variables were described as the means ± SD, whereas skewed distributed variables were described as median (25% and 75% interquartiles). Categorical variables were described as frequency (percentage). Log-transformations were applied to all variables with skewed distributions for the further analyses.

Comparisons between two subgroups were performed using unpaired t-tests for normally distributed variables, Mann-Whitney U tests for skewed distributions and Chi-squared tests for categorical variables. The correlation between glycaemic parameters and the QTc interval were analyzed by Pearson’s correlation test. Multiple linear regression analysis was conducted to investigate the independently association of glycaemic parameters and other metabolic factors with the QTc interval. A multiple logistic regression analysis model was also applied to investigate the associations of the second and third tertiles (T2 and T3) of SD-PPG with prolonged QTc interval relative to the first tertile (T1) based on the odds ratio (OR) and 95% confidence interval (95% CI). A *p*<0.05 was considered statistically significant. All analyses were processed using the SPSS19.0 (IBM SPSS Inc., USA).

## Results

### Clinical characteristics of the participants

The clinical characteristics of the total participants and the two subgroups (normal QTc interval versus prolonged QTc interval) are shown in Table 1. This study enrolled 2904 patients with type 2 diabetes, 2406 (82.9%) with normal QTc interval and 498 (17.1%) with prolonged QTc interval. The prevalence of prolonged QTc interval in our study was 17.1%. The distribution of QTc interval and glycaemic parameters are shown in S1 Fig. The average QTc interval of the total participants was 420±31 ms, that of the group with normal QTc interval was 402±24 ms, and that of the group with prolonged QTc interval was 458±20 ms. Age, ratio of females, SBP, diabetic duration, HOMA-IR and ratio of hypertension in the group with prolonged QTc interval were higher than those in the group with normal QTc interval, while BMI was lower than in the group with normal QTc interval. DBP, ratios of smoking, TC, TG, HDLC, LDLC and UA showed no differences between the two subgroups. Comparisons of medication use revealed that the group with prolonged QTc interval more frequently underwent insulin treatment than the group with normal QTc interval but that there were no differences in statins medication, lifestyle intervention, insulin-secretagogue or insulin-sensitizer use between the two subgroups. With regard to glycaemic parameters, the group with prolonged QTc interval tended to have greater M-FPG, M-PPG, SD-PPG and HbA1c than the group with normal QTc interval, but SD-FPG was comparable between the two subgroups.

**Table 1:** Clinical characteristics of the participants.

Variables	Total	QTc interval		*t/x*^*2*^	*p*
		≤440 ms	>440 ms		
n	2904	2406	498	–	–
Age (year)	56.1±13.5	55.3±13.5	55.4±12.7	–6.638	0.000
Female, n (%)	1380(47.5)	1030(42.8)	350(70.3)	124.9	0.000
BMI (kg/m^2^)	24.9±3.7	25.0±3.7	24.6±3.9	2.249	0.025
SBP (mmHg)	135±17	134±17	137±18	–3.393	0.001
DBP (mmHg)	80±10	80±10	80±11	–0.142	0.887
Diabetic duration (year)	4.0(1.5–9.0)	4.0(1.5–8.0)	4.0(1.5–8.0)	–	0.335
Hypoglycaemic treatments					
Lifestyle intervention alone, n(%)	312(10.7)	266(11.1)	46(9.2)	1.423	0.233
Insulin, n(%)	861(29.6)	668(27.8)	193(38.8)	23.90	0.000
Insulin-secretagogues, n(%)	1160(39.9)	980(40.7)	180(36.1)	3.619	0.057
Insulin-sensitizers, n(%)	1860(64.0)	1548(64.3)	312(62.7)	0.511	0.475
Hypertension, n(%)	1077(37.1)	840(34.9)	237(47.6)	28.42	0.000
Statins medication, n(%)	1071(36.9)	872(36.2)	199(40.0)	2.499	0.118
Smoking, n(%)	883(30.4)	720(29.9)	163(32.7)	1.535	0.215
TG (mmol/L)	1.61(1.02–2.58)	1.60(1.01–2.57)	1.63(1.07–2.61)	–	0.465
TC (mmol/L)	4.72±1.25	4.69±1.30	4.78±1.29	–0.617	0.517
HDLC (mmol/L)	1.07±0.28	1.07±0.28	1.08±0.29	–0.842	0.400
LDLC (mmol/L)	2.51±0.82	2.49±0.81	2.54±0.89	–0.933	0.351
UA (μmol/L)	285±101	286±98	282±113	0.618	0.536
HOMA-IR	2.58(1.55–4.14)	2.52(1.52–4.03)	2.91(1.69–4.51)	–	0.000
HbA1c (%)	8.23±1.25	8.15±1.23	8.60±1.28	–7.247	0.000
M-FPG (mmol/L)	7.47±0.97	7.43±0.95	7.68±1.03	–5.286	0.000
SD-FPG (mmol/L)	1.35±0.44	1.34±0.43	1.36±0.44	–0.437	0.662
M-PPG (mmol/L)	13.47±1.79	13.41±1.78	13.89±1.84	–5.431	0.000
SD-PPG (mmol/L)	2.11±0.68	2.05±0.65	2.40±0.76	–10.556	0.000
QTc interval (ms)	420±31	402±24	458±20	–48.633	0.000

QTc interval: heart rate-corrected QT interval; FPG: fasting plasma glucose; PPG: 2-hour postprandial plasma glucose; SD-FPG: standard deviation of FPG; SD-PPG: standard deviation of PPG; M-FPG: mean of FPG; M-PPG: mean of PPG.

### Relationships between glycaemic parameters and QTc interval

**Fig 1:**
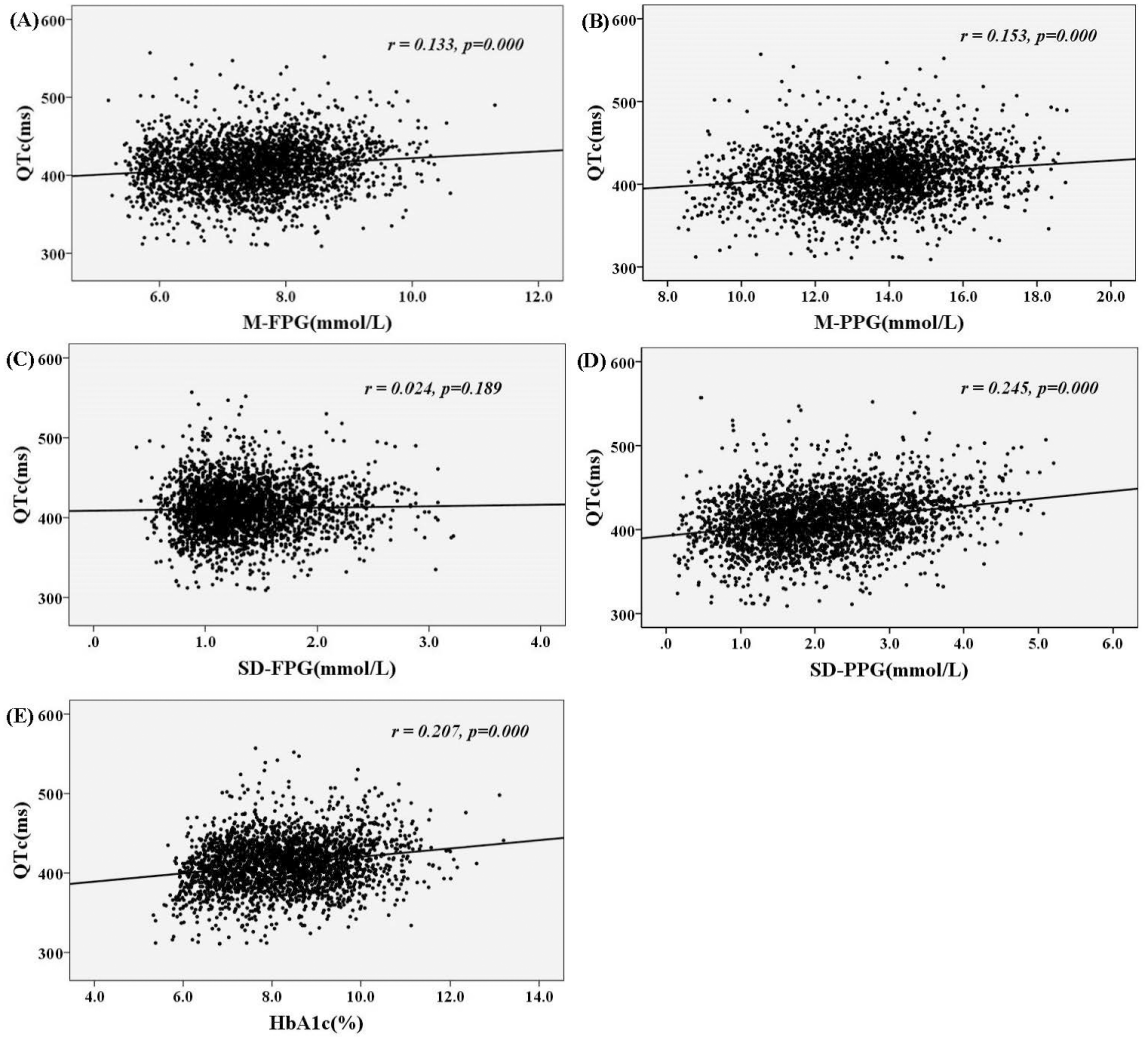
The relationships among glycaemic parameters (A: M-FPG, B: M-PPG, C: SD-FPG, D: SD-PPG, E: HbA1c) and QTc interval in the participants.

The correlations between glucose parameters and QTc interval are presented in Fig 1. The QTc interval was significantly positively correlated with M-FPG, M-PPG, SD-PPG and HbA1c (*r* = 0.133, 0.153, 0.245 and 0.207, respectively, *p* = 0.000), but not with SD-FPG (*r* = 0.024, *p* = 0.189).

### Multiple linear regression analysis with QTc interval as the dependent variable

The QTc interval was significantly correlated with M-FPG, M-PPG, SD-PPG and HbA1c based on an unadjusted test, and therefore, a multiple linear regression analysis was performed to establish which variables were independently associated with QTc interval. The independent factors included age, sex, SDP, DBP, BMI, diabetic duration, hypoglycaemic treatment, statins medication, hypertension, smoking, UA, TC, LDLC, HDLC, TG, HOMA-IR, HbA1c, M-FPG, M-PPG, SD-FPG and SD-PPG. After adjusting for the metabolic risk factors by the multiple linear regression analysis, SD-PPG (*β* = 0.208, *t* = 12.16 *p* = 0.000), M-PPG (*β* = 0.088, *t* = 2.69, *p* = 0.000) and HbA1c (*β* = 0.237, *t* = 10.16, *p* = 0.000) were found to be the major independent contributors for increased QTc interval. And M-FPG and SD-FPG were not independent risk factors for increased QTc interval. The independent contribution of SD-PPG for increased QTc interval was greater than M-PPG (Table 2).

**Table 2:** Multiple linear regression analysis with QTc interval as the dependent variable in participants.

Variables	*B*	*SE*	*β*	*t*	*p*
constant	288.006	10.478		27.488	0.000
Age	0.338	0.052	0.144	6.478	0.000
Female	15.816	1.226	0.253	12.898	0.000
BMI	–0.165	0.164	–0.020	–1.006	0.315
Diabetic duration	0.108	0.112	0.020	0.963	0.336
SBP	0.030	0.039	0.017	0.776	0.438
DBP	0.039	0.064	0.013	0.616	0.538
Hypertension	5.733	1.290	0.089	4.444	0.000
Statins medication	0.119	1.311	0.002	0.091	0.928
Lifestyle intervention	–6.298	2.669	–0.057	–2.359	0.018
Insulin	–0.461	1.844	–0.007	–0.250	0.803
Insulin-sensitizers	–1.962	1.401	–0.029	–1.400	0.162
Insulin-secretagogues	–2.272	1.636	–0.036	–1.388	0.165
Smoking	–1.299	1.397	–0.019	–0.930	0.353
TG	0.258	0.340	0.019	0.759	0.448
TC	–0.853	0.837	–0.034	–1.019	0.308
HDLC	1.797	2.307	0.016	0.779	0.436
LDLC	0.312	1.076	0.008	0.290	0.772
UA	0.024	0.006	0.076	3.750	0.000
HOMA-IR	2.264	0.805	0.057	2.812	0.005
HbA1c	6.043	0.595	0.237	10.161	0.000
M-FPG	1.795	1.222	0.054	1.468	0.142
M-PPG	1.572	0.585	0.088	2.687	0.007
SD-FPG	–1.489	2.285	–0.021	–0.651	0.515
SD-PPG	9.324	0.917	0.208	12.163	0.000

*B*, Regression coefficient; *SE*, Standard error; *β*, Standardized coefficient

### Proportion and odd ratios (ORs) of abnormally prolonged QTc interval according to SD-PPG tertiles

The proportion of abnormally prolonged QTc interval increased significantly from 10.9% to 14.2% to 26.6% from the first (T1) to second (T2) to third (T3) SD-PPG tertiles (*p* for trend = 0.000). Table 3 also shows the ORs of abnormally prolonged QTc interval according to the SD-PPG tertiles. Compared with participants in the first tertile of SD-PPG, the ORs of abnormally prolonged QTc interval for the second and third SD-PPG tertiles were 1.34 (95% CI, 1.03–1.76) and 2.96 (2.31–3.79), respectively. After adjusting via multiple logistic regression, the corresponding ORs of abnormally prolonged QTc interval for the T2 and T3 versus the T1 of SD-PPG were 1.15 (0.82–1.60) and 2.62 (1.92–3.57), respectively.

**Table 3:** Proportion and odd ratios (ORs) of abnormally prolonged QTc interval according to SD-PPG tertiles (95% CI).

SD-PPG tertiles	T1	T2	T3	*P* for trend
N	972	970	962	–
Abnormally prolonged QTc, n(%)	106(10.9)	137(14.1)	255(26.5)	0.000
Model 1	1- reference	1.34(1.03–1.76)	2.96(2.31–3.79)	0.000
Model 2	1- reference	1.42(1.08–1.88)	2.77(2.15–3.56)	0.000
Model 3	1- reference	1.43(1.06–1.91)	2.80(2.14–3.65)	0.000
Model 4	1- reference	1.15(0.83–1.61)	2.63(1.93–3.59)	0.000
Model 5	1- reference	1.15(0.82–1.60)	2.62(1.92–3.57)	0.000

Model 1: unadjusted model. Model 2: adjusted for age and sex. Model 3: additionally adjusted for diabetic duration, BMI, SBP, DBP, smoking, statins medication use and hypertension. Model 4: additionally adjusted for UA, TG, TC, HDLC, LDLC, HOMA-IR, HbA1c, M-FPG, M-PPG and SD-FPG. Model 5: additionally adjusted for lifestyle intervention and insulin, insulin-sensitiszers and insulin-secretagogues use.

## Discussion

In this study, we compared the association of long-term glycaemic variability versus average glucose with the QTc interval in a large-scale Chinese population with type 2 diabetes. Glycaemic variabilities were assessed by the standard deviations of SD-FPG and SD-PPG. The average glucose parameters were assessed by the mean FPG (M-FPG), the mean PPG (M-PPG) and HbA1c. The advantages of our study are as follows: first, the large Chinese population with type 2 diabetes presented a considerably high prevalence of prolonged QTc interval (>440 ms) with an incidence of 17.1%; second, in addition to sustained chronic hyperglycaemia assessed by M-PPG or current HbA1c, increased SD-PPG was a stronger independent risk factor for increased QTc interval in type 2 diabetes patients, whereas M-FPG and SD-FPG were not; third, compared with patients in the first SD-PPG tertile, those in the second and third SD-PPG tertiles were associated with increased risk for prolonged QTc interval, with multiple-adjusted ORs of 1.15 (95% CI, 0.82–1.60) and 2.62 (1.92–3.57), respectively.

### The glycemic variability and diabetic complications

Glycaemic variability is an important risk factor for diabetic complications. Glycaemic variability, as a component of glycaemic disorders, may have more deleterious effects than sustained chronic hyperglycaemia in the development of diabetic complications [22]. Monnier et al. [12] reported that short-term glycaemic variability exhibited a more specific triggering effect on oxidative stress than sustained hyperglycaemia, with activation of oxidative stress as one of the main mechanism chronic s leading to vascular complications. Several previous studies have observed that short-term glycaemic variability assessed by continuous glucose monitoring (CGM) plays a critical role in vascular endothelial dysfunction [23], diabetic neuropathy [24–25], and coronary artery disease [26] in type 2 diabetes patients. In addition, glycaemic variability accessed by CGM appeared to be of greater value than long-term sustained hyperglycaemia in the prognosis of macrovascular complications [27]. In addition to short-term glycaemic variability, some studies have also shown that long-term and day-to-day glycaemic variability can trigger oxidative stress and induce chronic inflammation [28, 29]. Long-term variation in FPG was a strong predictor of microvascular complications, ischaemic stroke and cardiovascular disease-related mortality in patients with type 2 diabetes [15–18]. Our present study showed that M-PPG, HbA1c and SD-PPG was each independently associated with prolonged QTc interval in type 2 diabetes, but the association of SD-PPG with prolonged QTc interval was stronger than the association with M-PPG or HbA1c. We did not observe a relationship between SD-FPG and the QTc interval. M-FPG was not an independent risk factor for prolonged QTc interval in type 2 diabetes, although it was correlated with QTc interval in the univariate analysis.

### The metabolic risk factors for increased QTc interval in type 2 diabetes

The prevalence of prolonged QTc interval in type 2 diabetes has been reported to range from 19% in Targher et al. [30] to 25.8% in Veglio et al. [9], and even to 44.1% in Ninkovic et al. [31]. In addition, our study showed that the prevalence of prolonged QTc interval in a large Chinese population with type 2 diabetes was 17.1%. Accumulating evidences had demonstrated that hyperglycaemia and coexisting confounders in diabetes may promote the increases in QTc interval. Increased QTc interval may be related to cigarette smoking [32], obesity [4], nonalcoholic fatty liver disease [30], hypertension [33], dyslipidaemia [6], uric acid (UA) [34], insulin resistance and its associated hyperinsulinaemia [35], glycaemic status [36], coronary artery disease, carotid intima media thickness, diabetic neuropathy, diabetic retinopathy, and diabetic cardiomyopathy [1, 9, 37]. In the present study, hypertension, UA and nonmodifiable factors including age and sex were observed to be independently associated with increased QTc interval. In addition, increased SD-PPG and M-PPG were independent risk factor for increased QTc interval in type 2 diabetes patients, whereas M-FPG and SD-FPG were not. And the independent contribution of SD-PPG for increased QTc interval was greater than M-PPG. Strategies targeting to long-term variability of postprandial glucose(SD-PPG) may provide therapeutic implications to improve prolongation of QTc interval and its associated prognosis of patients with type 2 diabetes.

### The possible mechanisms linking glycaemic variability, as assessed by the calculation of SD-PPG, and QTc interval

Activation of oxidative stress by hyperglycaemia plays an essential role in the pathogenesis of diabetic hearts, including atherosclerosis and cardiomyopathy [38, 39]. Brownlee et al. [40] demonstrated that the process of superoxide overproduction induced by hyperglycaemia in the mitochondrial electron-transport chain, which was an upstream and central event in four main damage pathways of diabetic complications involving increased polyol pathway flux, increased intracellular formation of advanced glycation end products (AGEs), activated protein kinase C (PKC) and nuclear factor kB (NF-kB), and increased flux through the hexosamine pathway. The QTc interval reflects the total time taken for ventricular myocardial depolarization and repolarization, and metabolic, morphological, functional and structural abnormalities of the myocardium may facilitate the increase in QT interval. The oxidative stress induced by hyperglycaemia may alter myocardial structure and function through multiple damage pathways [41], which in turn could increase the QT interval. For example, oxidative stress may play a predominant role in sub-cellular remodelling, impaired calcium homeostasis and subsequent left ventricular hypertrophy [42]. Defective calcium inactivation could also directly cause long QT interval [43]. Glycaemic variability could produce a more specific triggering effect on oxidative stress than chronic hyperglycaemia [12]. In addition, both acute and long-term glycaemic variability can induce oxidative stress and chronic inflammation [29]. SD-PPG could estimate long-term glycaemic variability based on a standard deviation of more than 4 times the PPG level in the 1 year preceding enrolment. SD-PPG was independently associated with increased QTc interval. Therefore, SD-PPG may be a potential marker for predicting prolonged QTc interval.

### Limitations

Some limitations of the present study should be addressed. First, the study based on a cross-sectional observational data and may not prove the existence of a causal link between the SD-PPG and the QTc interval. Prospective follow-up studies are needed to demonstrate the causal association. Second, our findings were based on a Chinese Han population and needed to be replicated in other populations. Third, we did not explore chronic glycaemic variability assessed by SD-PPG in relation to the markers of oxidative stress, inflammation and vascular dysfunction. Fourth, glycaemic variability in our study was not monitored by a CGM system, which could detect glycaemic variability in more detail than traditional methods of multiple glucose determinations [44, 45]. In the present, CGM could only record glycaemic profiles for a short term (3–7 days) because of the effectiveness of glucose sensors, which may attenuate its utility in the long-term applications. Additionally, CGM is costly and is hard to be applied in a large-scale study. The SD-PPG may be a simple but robust parameter for estimating chronic glycaemic variability.

### Conclusions

In summary, the increased long-term variability of PPG, in addition to long-term postprandial hyperglycaemia and current glycaemic control assessed by HbA1c, is a strong independent risk factor for increased QTc interval in patients with type 2 diabetes.

## Supporting information

S1 STROBE Checklist The STROBE checklist for the cross-sectional study. STROBE: Strengthening the Reporting of Observational studies in Epidemiology.
(DOCX)

S1 Fig The distribution of QTc interval(A), M-FPG(B), M-PPG(C), SD-FPG(D), SD-PPG(E) and HbA1c(F). M-FPG: mean of fasting plasma glucose; M-PPG: mean of postprandial plasma glucose; SD-FPG: standard deviation of fasting plasma glucose; SD-PPG: standard deviation of postprandial plasma glucose;
(TIF)
