# Supplementary figures and images for: The association of long-term glycaemic variability versus sustained chronic hyperglycaemia with heart rate-corrected QT interval in patients with type 2 diabetes
Source: PLoS One. 2017 Aug 28;12(8):e0183055. doi: 10.1371/journal.pone.0183055 (PMC5573287; doi:10.1371/journal.pone.0183055)

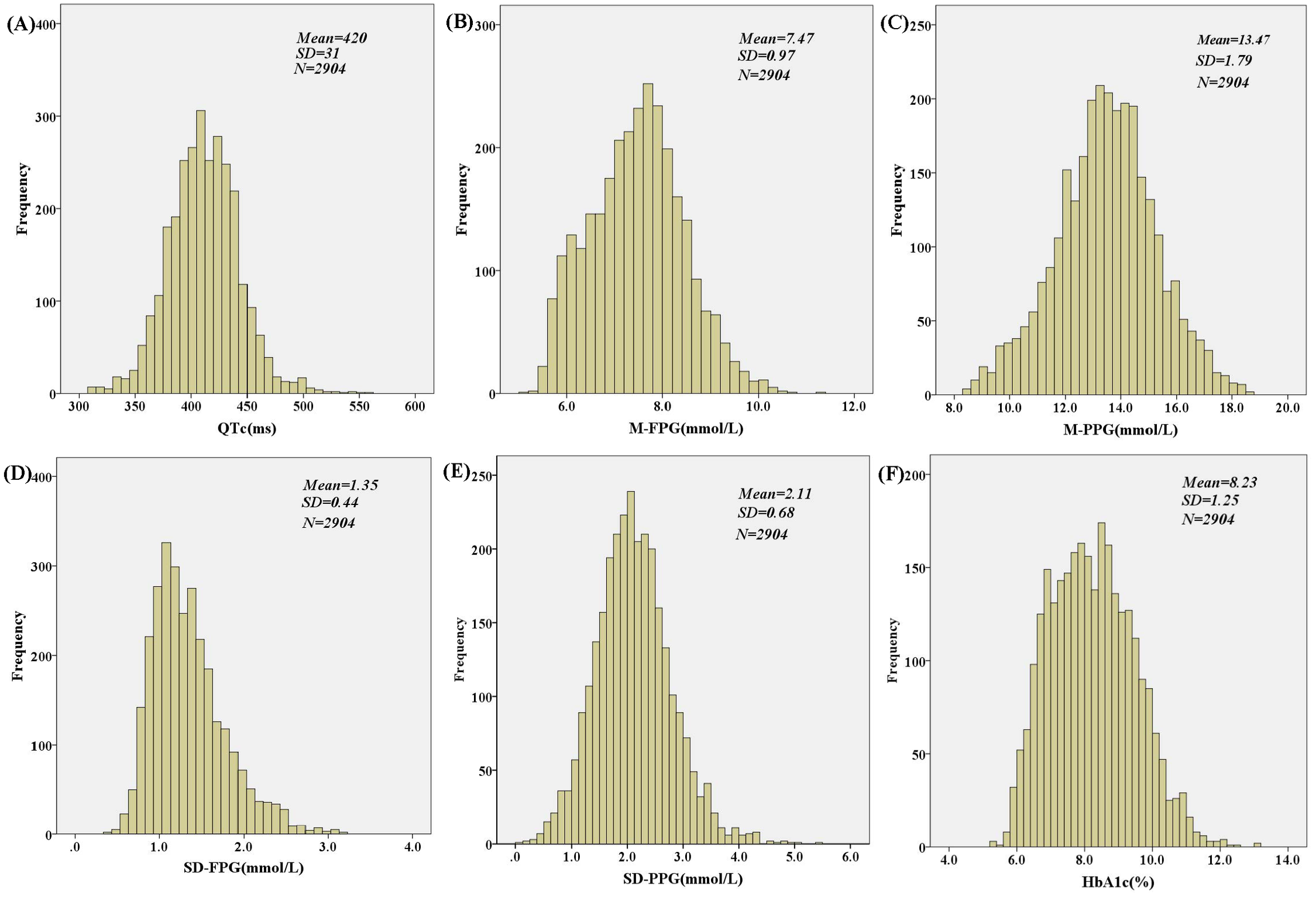

Supplement: S1 Fig — M-FPG: mean of fasting plasma glucose; M-PPG: mean of postprandial plasma glucose; SD-FPG: standard deviation of fasting plasma glucose; SD-PPG: standard deviation of postprandial plasma glucose; (TIF) [file pone.0183055.s002.tif]
